# Line-Monitoring, Hyperspectral Fluorescence Setup for Simultaneous Multi-Analyte Biosensing

**DOI:** 10.3390/s111110038

**Published:** 2011-10-25

**Authors:** Zhiyi Liu, Heng Shi, Le Liu, Sunan Deng, Yanhong Ji, Suihua Ma, Hui Ma, Yonghong He

**Affiliations:** 1 Laboratory of Optical Imaging and Sensing, Graduate School at Shenzhen, Tsinghua University, Shenzhen 518055, China; E-Mails: liuzhiyi07@mails.tsinghua.edu.cn (Z.L.); shiheng_2008@126.com (H.S.); liule99@mails.tsinghua.edu.cn (L.L.); dengsunan89@163.com (S.D.); annie.shma@gmail.com (S.M.); mahui@tsinghua.edu.cn (H.M.); 2 MOE Key laboratory of Laser Life Science & Institute of Laser Life Science, South China Normal University, Guangzhou 510631, China; E-Mail: jiyh@scnu.edu.cn (Y.J.)

**Keywords:** hyperspectral fluorescence detection, microfluidic system, biosensor

## Abstract

Conventional fluorescence scanners utilize multiple filters to distinguish different fluorescent labels, and problems arise because of this filter-based mechanism. In this work we propose a line-monitoring, hyperspectral fluorescence technique which is designed and optimized for applications in multi-channel microfluidic systems. In contrast to the filter-based mechanism, which only records fluorescent intensities, the hyperspectral technique records the full spectrum for every point on the sample plane. Multivariate data exploitation is then applied to spectra analysis to determine ratios of different fluorescent labels and eliminate unwanted artifacts. This sensor is designed to monitor multiple fluidic channels simultaneously, providing the potential for multi-analyte biosensing. The detection sensitivity is approximately 0.81 fluors/μm^2^, and this sensor is proved to act with a good homogeneity. Finally, a model experiment of detecting short oligonucleotides has demonstrated the biomedical application of this hyperspectral fluorescence biosensor.

## 1. Introduction

Fluorescence imaging, due to its high sensitivity and selectivity, has become one of the most popular analytical tools for detecting biomolecular interactions [1–3]. Nowadays, fluorescence imaging is required in a number of fields, especially in biological, medical and food industries [4,5]. Conventional fluorescence scanners are filter-based, with multiple filters employed to distinguish different fluorescent labels [6]. There are some disadvantages for this filter-based mechanism. Firstly, when multiple fluorescent labels are needed, the chosen labels should have minimal overlap of their emission spectra, otherwise, it is difficult to identify various labels using only filters. Secondly, it poses challenges for filter-based scanners to determine whether the measured data have been corrupted by some extraneous emission sources. For instance, the contamination induced by fabrication chemistry as well as the emissions from the substrates [7] may affect the determination of gene or protein expressions [8]. To overcome these problems, we have previously developed a quasi-confocal, parallel scan hyperspectral fluorescence imaging system, which has been successfully applied to microarray analysis [9]. The hyperspectral fluorescence approach works by recording full spectra of all the points on the sample plane via a spectrometer [8–10]. Multivariate data exploitation is then employed for analysis of spectral information to accurately determine ratios of different emission sources [11,12]. As a result, the restrictions on the allowable fluorescent labels are reduced, and the detection accuracy is enhanced by removal of emissions of the contamination and substrates, exhibiting advantages in multi-label strategies.

Microfluidic systems are of great significance and have been widely employed for biochemistry applications [13–15]. A great many microfluidic systems only provide mono-channel detection with a single flow cell [16,17]. However, in some cases it is necessary to carry out multi-analyte sensing, while the mono-channel detection is difficult to do so. Moreover, a fixed point of the flow cell is conventionally monitored in a fluidic protocol. However, the biomolecular interactions vary among different points, and the information of a fixed point can hardly provide a comprehensive analysis of biological events. In order to carry out multi-analyte biosensing, microfluidic systems with multiple channels have been developed [18,19], offering the potential for high-throughput and multi-channel biomedical research.

In this work we present a line-monitoring, hyperspectral fluorescence setup which is optimized for applications in multi-channel microfluidic systems. The laser light is focused to a line to cover all the channels of the microfluidic system for fluorescence excitation, and thus multiple channels can be monitored simultaneously. Moreover, a small region of the fluidic channel is scanned and the final output is an average of signals of the scanned region. In this way we acquire information of an area instead of a fixed point, reducing measurement errors that might occur. The performance of this sensor is characterized, and its biomedical application has been demonstrated by a model experiment detecting the hybridization of short oligonucleotides.

## 2. Methods and Materials

### 2.1. Reagents

Throughout our study, the used synthesized oligonucleotides are as follows:

Escherichia coli:
Probes: 5′ (SH)-ACG GTT ACC TTG TTA CGA CTT-3′,
Complementary oligonucleotides:
5′-TGC CAA TGG AAC AAT GCT GAA-3′ (Cy5),
5′-TGC CAA TGG AAC AAT GCT GAA-3′ (Dylight 680).
β-hemolytic *Streptococcus*:
Probes: 5′ (SH)-ACC GTC ACT TGG TGG ATT-3′,
Complementary oligonucleotides:
5′-TGG CAG TGA ACC ACC TAA-3′ (Dylight 680).

In this work, we use magnetron sputtering gold films as substrates of microfluidic systems, and all the DNA probes are thiolated for covalent bonding to substrates. Fluorophores of Cy5 (absorption/emission: 649/667 nm) and Dylight 680 (absorption/emission: 680/715 nm) are used as fluorescence tags and labeled to complementary oligonucleotides. Both DNA probes and complementary genes are purchased from TaKaRa Biotechnology (Dalian, China). Absolute ethanol is purchased from Meryer (Shanghai, China), and deionized water is prepared by ourselves.

### 2.2. Sensor Instrumentation

**Figure 1.**
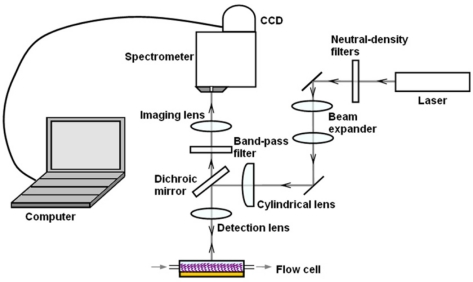
Schematic representation of this line-monitoring, hyperspectral fluorescence setup.

Figure 1 shows the layout of this system. A He-Ne laser with a wavelength of 632.8 nm and a power of 12.5 mW is used as the light source for excitation of fluorescence signals. Neutral-density filters are used to adjust laser intensities to fit experimental conditions. Then the laser light is expanded by a beam expander, and focused by a cylindrical lens (*f* = 30 mm) for the desired line light on the surface of the dichroic mirror, which is characterized by high reflectivity at the laser wavelength and high transparency at longer wavelengths. A detection lens (*f* = 50 mm) focuses the laser light to the microfluidic system mounted on an *x*-*y* scan stage. Detection lenses with different focal lengths can be chosen according to the detection purpose. In this work the detection lens used offers a line beam long enough to cover all the fluidic channels of the microfluidic system. We design and fabricate a 5-channel microfluidic system which allows the continuous delivery of sample solutions through these channels at a constant throughput speed of 20 μL/min unless otherwise noted.

Fluorescence excited by the focused line light is collected by the detection lens and directed by the dichroic mirror. A band-pass filter (with 85% transmission at *λ* = 700 nm, bandwidth = 75 nm) is used to further eliminate the scattered and reflected laser light, and an imaging lens (*f* = 50 mm) is used to image the pattern of the excited line region onto the entrance slit of a homemade spectrometer. The width of the entrance slit is 12 μm, which is set in accordance to the width of the focused line light. This spectrometer offers a high spectral resolution of 0.2 nm [9], which is good in separating spectra in most multicolor assays. A CCD array (Canon 500D) is used to capture the spectrally resolved images, and a personal computer carries out further data analysis.

### 2.3. Data Analysis

**Figure 2.**
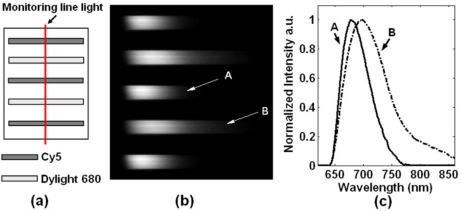
Spectral data analysis. **(a)** Design of the sensing model. **(b)** The dispersed image corresponding to the monitored line region. **(c)** Normalized intensity curves of marked rows A and B.

To demonstrate the data processing, we designed a sensing model for fluorescence imaging, as shown in Figure 2(a). There are five fluidic channels containing different solutions of fluorophores. The 1st, 3rd and 5th channels contain solutions of Cy5, while the 2nd and 4th channels contain solutions of Dylight 680. Figure 2(b) is a spectrally resolved image captured by CCD, corresponding to the illuminated line region in Figure 2(a). In this image, each row represents the spectrum of a certain point of the line region. The normalized intensity curves of two marked rows A and B are shown in Figure 2(c). It is illustrated that the spectra of these two dyes are highly overlapped, while the hyperspectral mechanism distinguishes these two fluorophores by spectral resolving.

## 3. System Characterization

### 3.1. Detection Sensitivity

The detection sensitivity of a fluorescence imaging system is calculated as follows [20,21]:

(1) $$S_{d}=D_{s}/\mathrm{SNR}$$

(2) $$D_{s}=V_{p}\times M_{p}\times N_{A}/A_{o}$$

(3) $$\mathrm{SNR}=\left(n_{s}-n_{b}\right)/\sigma_{b}$$

**Figure 3.**
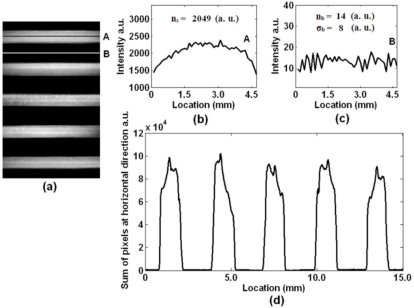
Performance of this biosensor. **(a)** Fluorescence image of a small region (∼4.5 mm) of the fluidic system. **(b)** Intensity curve of marked row A. **(c)** Intensity curve of marked row B. **(d)** Gray sum of all pixels at horizontal direction.

In Equation (1), *S_d_* is the detection sensitivity, *D_s_* the sample density and *SNR* the signal-to-noise ratio. The *D_s_* and *SNR* calculations are shown in Equations (2) and (3), respectively. In Equation (2), *N_A_* is Avogadro’s constant, *V_p_* the extracted volume of solution, *M_p_* the concentration of fluorophores with a unit of μmol/L, and *A_o_* the area of the solution on the sensing plane; thus the unit of sample density is the number of fluorescence molecules per square micro (fluors/μm^2^). In this test, Cy5 solution with a sample density of approximately 206 fluors/μm^2^ is applied to each fluidic channel. In order to demonstrate the sensitivity of this sensor, we scan a small region (∼4.5 mm) of these five channels, with the scanning image shown in Figure 3(a). This scanning image is acquired by analyzing and combining all the spectrally resolved images obtained during optical scan. Equation (3) shows the calculation of signal-to-noise ratio [22], where *n_s_* is the average fluorescence intensity, *n_b_* the average intensity of background, and *σ_b_* the standard deviation of background. To illustrate the fluorescence intensity and background noise level, intensity graphs of marked rows A and B are plotted, as shown in Figure 3(b,c), respectively. The values of *n_s_*, *n_b_* and *σ_b_* are given in these graphs, and an *SNR* of 254 is acquired. According to Equation (1), the sensitivity of this sensor is approximately 0.81 fluors/μm^2^, which is adequate and good for fluorescence acquisition in most cases.

### 3.2. Homogeneity

Here we test the detection homogeneity of this sensor with the scanning image shown as Figure 3(a). If this image is described as a data matrix *S*_(_*_m_*_,_*_n_*_)_, where n and m are pixel numbers along the vertical direction and along the horizontal direction, respectively, then the gray sum of all pixels at horizontal direction is as follows:

(4) $$\mathrm{col}(y)=\sum_{x=1}^{m}S(x,y),x=1,2,\cdots ,m$$

The calculation results are shown in Figure 3(d). The intensity peaks in Figure 3(d) are 9.9, 10.2, 9.3, 9.7 and 9.1 (10^4^ a.u.), with a maximum fluctuation of 11%. It is illustrated from the calculation results that this sensor exhibits a good homogeneity.

## 4. Biomedical Experiments

**Figure 4.**
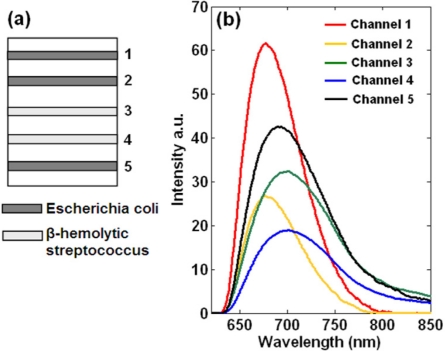
Biomedical application of this setup for detecting short oligonucleotides. **(a)** Different kinds of DNA probes immobilized on the substrate corresponding to different channels. **(b)** Setup response to samples with different oligonucleotides and fluorescent labels.

In order to demonstrate the biomedical application of this line-monitoring, hyperspectral fluorescence biosensor, we designed a model experiment to detect short oligonucleotides for identification of bacteria using multi-channel microfluidic systems. Figure 4(a) shows the preparation of DNA probes. As can be seen, the 1st, 2nd and 5th channels immobilize *Escherichia coli* probes (5′ (SH)-ACG GTT ACC TTG TTA CGA CTT-3′), and the other two channels immobilize β-hemolytic streptococcus probes (5′ (SH)-ACC GTC ACT TGG TGG ATT-3′). Then five kinds of solutions that contain different fluorescence labeled complementary oligonucleotides are flowed through these fluidic channels at a flow-rate of 20 μL/min by means of a peristaltic pump. The sample solutions used corresponding to each channel are as follows: 1 μmol/L Cy5 labeled *Escherichia coli* oligonucleotides (channel 1), 0.5 μmol/L Cy5 labeled *Escherichia coli* oligonucleotides (channel 2), 2 μmol/L Dylight 680 labeled β-hemolytic streptococcus oligonucleotides (channel 3), 1 μmol/L Dylight 680 labeled β-hemolytic *Streptococcus* oligonucleotides (channel 4), and a mixture of 0.5 μmol/L Cy5 labeled *Escherichia coli* oligonucleotides and 2 μmol/L Dylight 680 labeled *Escherichia coli* oligonucleotides (channel 5). These synthesized oligonucleotides present in sample solutions are complementary to the substrate bound DNA probes. The excitation efficiency of Dylight 680 by a He-Ne laser is weaker than that of Cy5, and thus the applied concentration of Dylight 680 labeled sample solutions is higher than that of Cy5 labeled solutions. After the hybridization process, fluidic channels are rinsed thoroughly by deionized water to remove unbound oligonucleotides, and then ready for optical scan by this hyperspectral fluorescence setup.

Figure 4(b) shows the detection results with one spectral curve corresponding to each channel. Every spectral curve is acquired by two averaging processes. Firstly, a small region (∼1 mm) of these fluidic channels (∼0.8 mm in width per channel) is scanned, and all the scanning images are averaged. Secondly, all the rows corresponding to a channel are averaged. Finally we acquire a spectral curve representing each fluidic channel, by which a wealth of information can be obtained. The sample concentration and hybridization efficiency are demonstrated by the relative fluorescence intensity, and the biochemical interactions can be revealed via the spectral characterization due to the specificity of each fluorescent spectrum. The curves corresponding to the 1st and 2nd channels exhibit identical spectra due to the same fluorescent labels, except that the fluorescence intensities vary from each other because of different sample concentrations. The similar results are observed in the 3rd and 4th channels.

**Figure 5.**
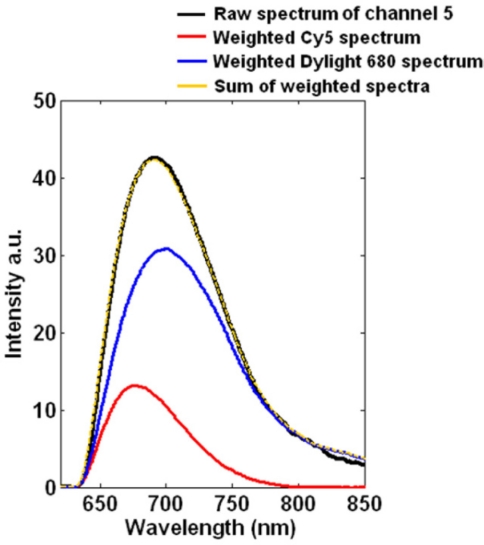
Detailed analysis of the raw spectrum of channel 5. Black curve: the raw spectrum; red curve: the weighted spectrum for Cy5; blue curve: the weighted spectrum for Dylight 680; yellow curve: the calculated spectrum as a sum of these two weighted spectra.

In the 5th channel, both Cy5 and Dylight 680 labeled complementary *Escherichia coli* oligonucleotides are present in the flowing sample solutions. Thus the acquired spectrum of channel 5 is different from absolute Cy5 or Dylight 680 spectrum. We apply multivariate data exploitation to the raw spectrum of channel 5 to determine ratios of each dye, as shown in Figure 5. The black curve represents the raw spectrum, and the red and blue curves represent the weighted Cy5 and Dylight 680 spectra, respectively, which are calculated by multivariate data analysis. The yellow curve is a sum of the weighted Cy5 and Dylight 680 spectra. As can be seen from Figure 5, the sum curve closely corresponds to the raw spectrum, revealing that the multivariate algorithm that we have developed can successfully model the hyperspectral stack using linear admixtures of these two dyes. Besides distinguishing different fluorescent tags, the significance of this experiment in channel 5 is that our system can also manage to identify and eliminate unwanted artifacts, e.g., the fluorescence emissions from the substrate or contamination, by spectrally resolving the overall raw spectra.

## 5. Conclusions

In this work we propose a line-monitoring, hyperspectral fluorescence biosensor designed and optimized for applications in multi-channel, microfluidic systems. By recording the fluorescence spectra instead of intensities, the hyperspectral technique enhances the detection accuracy by identification and elimination of some unwanted artifacts. Moreover, this sensor is able to detect multiple fluidic channels simultaneously, providing the potential for concurrent multi-analyte biosensing. The output of this sensor, behaving as a spectral curve, shows both relative intensity and the spectrum characterization of corresponding fluorescent labels, and thus the concentration of the sample solution as well as the information related to the biomolecules can be obtained. The sensitivity of this biosensor is better than 1 fluors/μm^2^, and its homogeneity is proved good. On account of these features, we believe that this line-monitoring, hyperspectral fluorescence biosensor has the potential for providing high-throughput and high-accuracy analysis of biochemical interactions.
